# Editorial: Post Operative Radiation Treatment for Prostate Cancer

**DOI:** 10.3389/fonc.2022.959436

**Published:** 2022-07-05

**Authors:** I. Latorzeff, P. Sargos, F. Saad

**Affiliations:** ^1^ Department of Radiation Oncology, Clinique Pasteur, Toulouse, France; ^2^ Department of Radiation Oncology, Institut Bergonié, Bordeaux, France; ^3^ Centre Hospitalier de l'Université de Montréal, Université de Montréal, Montréal, QC, Canada

**Keywords:** prostate cancer, post operative radiation therapy, adjuvant treatment, salvage treatment, functional imaging

Radical Prostatectomy (RP) is a standard treatment for localized prostate cancer (PCa). Approximately 30% of patients treated with RP for prostate cancers will experience biochemical recurrence (BCR) (1). Post-operative radiation therapy (RT) can be either offered immediately after the surgery in case of aggressive pathological featuresdefined adjuvant treatment or be proposed for patients experiencing BCR as an earlysalvage strategy (2–5). Thereby the management of the post-operative BCR is most often based on salvage radiotherapy (SRT) with or without the addition of hormone therapy (HT) (3–5). The indications for SRT +/- HT are established in the setting of a rising PSA level, after a period where an undetectable PSA was achieved (2, 3). However, immediate SRT in case of detectable PSA immediately after RP, but also SRT when a macroscopic recurrence in the prostate bed is shown by functional images, are two issues which treatment options and prognosis are still unclear (4, 5). The present edition of Frontiers in Oncology focuses on the need to improve the outcome of men with high risk or recurrent prostate cancer. This edition also focuses on the need for multi-disciplinary collaboration to achieve this important overall oncological objective. The range of topics in this collection features contributions to summarize the best evidence for post operative radiation therapy and highlight emerging advances in this setting. Manuscripts will target the need to optimize detection of relapse with the use of new generation imaging in the idea to improve outcome by early identification and treatment of distant sites of recurrence (Renard-Penna et al.; Cailleteau et al.; Gonzalez-Moya et al.). The key question wether or not adjuvant RT still remains a routine indication of treatment or should it be replaced in all situation by early SRT will be discuss as well (Giraud et al.). Because combinations of ADT with other systemic therapies add to the complexity of managing these patients presenting BCR, SRT encompass an evolving landscape of options and emerging concepts (radiation dose, timing of SRT, addition of next generation HT or chemotherapy) will be highlighted (le Guevelou et al.).

We hope that this collection of articles contributes to the need for interdisciplinary collaborations on this topic and will give radiation oncologist an ideal overview on how to manage this patients usually discussed and/or referred to our institutions. Finally, with the help of several experts, our goal was to enlarge the field of post-operative RT to a better personalized approach.

## Author Contributions

All authors listed in the editorial have participated to a substantial, direct or intellectual contribution to the collection of articles and they approved it for publication.

## Conflict of Interest

The authors declare that the research was conducted in the absence of any commercial or financial relationships that could be construed as a potential conflict of interest.

## Publisher’s Note

All claims expressed in this article are solely those of the authors and do not necessarily represent those of their affiliated organizations, or those of the publisher, the editors and the reviewers. Any product that may be evaluated in this article, or claim that may be made by its manufacturer, is not guaranteed or endorsed by the publisher.
